# Draft genome sequence of the naphthalene-degrading bacterium *Rhodococcus pyridinivorans* RA1 isolated from an industrial soil sample in Mosul, Iraq

**DOI:** 10.1128/mra.00611-24

**Published:** 2025-03-19

**Authors:** Ahmed Y. Al-Shiti, Rayan M. Faisal, Talal S. Salih, Gerben J. Zylstra

**Affiliations:** 1 College of Pharmacy, University of Ninevah, Mosul, Iraq; 2 Department of Biology, College of Science, University of Mosul, Mosul, Iraq; 3 Department of Medical Physics, College of Science, University of Mosul, Mosul, Iraq; 4 Department of Biochemistry and Microbiology, School of Environmental and Biological Sciences, Rutgers University, New Brunswick, New Jersey, USA; California State University San Marcos, San Marcos, California, USA

**Keywords:** *Rhodococcus*, naphthalene, biodegradation

## Abstract

*Rhodococcus pyridinivorans* RA1 was isolated for the ability to grow on naphthalene from an industrial site near Mosul, Iraq. The draft genome is 5,419,924 bp with a GC content of 67.5%.

## ANNOUNCEMENT

Naphthalene with two aromatic rings is the simplest polycyclic aromatic hydrocarbon (PAH) and is often used as a model for PAH degradation (1, 2). We are interested in the genetic diversity of naphthalene-degrading bacteria from diverse habitats around the world. A scoop of surface soil from an industrial area near Mosul, Iraq (36°27'18.7"N 43°05'31.5"E) was obtained in August 2014. Naphthalene-degrading bacteria were isolated by adding 1.0 g of soil to 50 mL of mineral salts basal (MSB) medium (3), with 3 mM naphthalene and incubated for 5 days at 30°C on a rotary shaker. Following one subculture on the same medium, the cells were plated on LB agar (4). Colonies were streaked for purity on MSB agar with naphthalene, and one isolate was designated as strain RA1.

RA1 genomic DNA was extracted using the DNeasy UltraClean Microbial Kit (QIAGEN, Germantown, MD). The RA1 genome was sequenced by MicrobesNG at the University of Birmingham, UK, using an Illumina HiSeq 2500 with a 250 bp paired-end protocol (Illumina, San Diego, USA) from a DNA library prepared using the Nextera XT Library Prep Kit (Illumina, San Diego, USA) using default parameters except that the input DNA was increased 2-fold, and the PCR elongation time was increased to 45 s. Reads (592, 578) were processed with Trimmomatic version 0.30 (5), with a sliding window quality cutoff of Q15. *De novo* assembly of the 567,907 processed paired-end reads was with SPAdes 3.5 (6), applying settings of k-mer length of 21, 33, and 55 and enabling single-cell mode as recommended for high GC data sets. QUAST 2.3 software (7) was used to generate assembly statistics. The genome was annotated with the NCBI Prokaryotic Genome Annotation Pipeline (PGAP) version 6.3 (8–10). The SEED tool (11) was used to predict functional genes in subsystem categories. Default parameters were used in the laboratory and computational protocols except where otherwise noted above.

The draft genome sequence of RA1 with 30× coverage is 5,419,924 bp with a GC content of 67.5% distributed within 132 contigs with an N50 value of 122,361 bp and with the largest contig 283,648 bp. PGAP annotated a total of 5,053 CDSs including 4,899 protein coding genes, 54 tRNAs, and 11 rRNAs (five 5S, five 16S, and one 23S). The SEED tool predicted 112 genes for the metabolism of aromatic compounds including genes encoding eight aromatic Rieske dioxygenases and nine *meta*-aromatic ring cleavage enzymes demonstrating that RA1, like many other rhodococci (12), is a versatile aromatic hydrocarbon degrader.

The Type Strain Genome Server (TYGS) (13) was used to infer the whole-genome-based phylogenetic tree of RA1 utilizing the Genome BLAST Distance Phylogeny (GBDP) method (14) using default settings and the dendrogram inferred with the FastME 2.0 program (15). The genome-to-genome distance (GGDH) bioinformatics tool (16) was used to measure *in silico* DNA-DNA hybridization (isDDH) values. The best matches to the RA1 genome are the type strains *R. pyridinivorans* DSM 44555 (accession NZ_LRRI00000000.1) and *R. biphenylivorans* TG9 (accession NZ_CP022208.1), with isDDH values of 87.4% and 83.5%, respectively.

## Data Availability

The draft genome sequence of *Rhodococcus* RA1 was deposited in GenBank under accession number JAPDNN000000000. BioProject, BioSample, and SRA (trimmed reads) accession numbers are PRJNA895202, SAMN31496625, and SRX24715778, respectively.
